# Gemcitabine and Rapamycin Exhibit Additive Effect against Osteosarcoma by Targeting Autophagy and Apoptosis

**DOI:** 10.3390/cancers12113097

**Published:** 2020-10-23

**Authors:** Takashi Ando, Jiro Ichikawa, Taro Fujimaki, Naofumi Taniguchi, Yoshihiro Takayama, Hirotaka Haro

**Affiliations:** Department of Orthopeadic Surgery, Yamanashi University School of Medicine, Yamanashi 409-3898, Japan; jiro@yamanashi.ac.jp (J.I.); tfujimaki@yamanashi.ac.jp (T.F.); naofumit@yamanashi.ac.jp (N.T.); ytakayama@yamanashi.ac.jp (Y.T.); haro@yamanashi.ac.jp (H.H.)

**Keywords:** gemcitabine, rapamycin, autophagy, apoptosis, angiogenesis

## Abstract

**Simple Summary:**

Osteosarcoma is the most common solid cancer of the bone. Unfortunately, the expected outcome for patients with this and similar types of cancers has changed little over the last 20 years. One important need is to develop new therapies that overcome the development of drug resistance in osteosarcoma cells. The drug gemcitabine (Gem) can kill osteosarcoma cells and prevent them from growing and spreading. Another drug, rapamycin (Rapa), can also kill osteosarcoma cells, but does so in a different way. Here, we investigated whether a combination of Rapa and Gem would exhibit better osteosarcoma treatment efficiency than Rapa and Gem monotherapies. We found that this drug combination was very effective at killing osteosarcoma cells, both in cell cultures and in mice, and the combinatorial treatment was better than treatment with a single agent. Therefore, combinatorial therapy with Gem and Rapa may be an effective approach for treating drug resistant osteosarcoma cells.

**Abstract:**

The overall prognosis for sarcoma-based cancer patients has remained largely unchanged over the past 10 years. Because there is no effective anticancer drug for patients with chemoresistant osteosarcoma (OS), novel approaches are needed to improve the prognosis. Here, we investigated whether rapamycin (Rapa) could enhance the anti-tumor effects of gemcitabine (Gem) in OS. Gem dose-dependently killed the OS cells, but exhibited much lower cytotoxicity on osteoblasts. Treatment with a combination Gem and Rapa was much more effective than that of either single agent with respect to reducing cell viability, cell invasion, cell migration, and vascular endothelial growth factor production in vitro. Moreover, the combination of these agents suppressed tumor growth, angiogenesis, and lung metastasis in allograft and xenograft murine models of OS with minimal adverse effects. Overall, the combination therapy prolonged the overall survival of tumor-bearing mice. Mechanistically, Gem induced apoptosis and increased the levels of cleaved caspases, while Rapa induced autophagy and microtubule-associated protein light chain 3 (LC3)-I/LC3-II expression both in vitro and in vivo. Our findings suggest that chemotherapy using Gem combined with Rapa may be a novel and promising therapeutic approach for the treatment of OS.

## 1. Introduction

Osteosarcoma (OS) is the most frequent primary solid malignancy of the bone and is associated with a high degree of malignancy, early metastasis, rapid progression, and poor prognosis. The incidence of OS is relatively low at 2–3/million/year; however, most patients are children/adolescents (<20 years of age) [1,2,3]. For OS, local therapy alone is insufficient. More than 80% of all patients with localized disease develop lung metastases and die when chemotherapy is not included as part of a multidisciplinary treatment [4]. Combination chemotherapy with methotrexate, doxorubicin, cisplatin, and ifosfamide is primarily used for treating patients with OS, and currently the 5-year survival rate has increased to 65% [5]. However, 40% of the patients with OS are insensitive to chemotherapy and their 5-year survival rate is only 5–20% [6]. Complications and fatal toxicities limit the use of chemotherapeutic agents. The overall prognosis of patients with sarcoma-based cancers has changed little in the last 20 years. No new drug with a high response rate has been developed and it remains difficult to improve prognosis using conventional chemotherapeutic regimens. Thus, an innovative strategy for overcoming OS drug resistance is urgently needed.

Gemcitabine (Gem; 2′,2′-difluoro 2′-deoxycytidine, dFdC) is the second cytidine analog developed after cytosine arabinoside (Ara-C). Currently, Gem is used as an agent for the treatment of patients with pancreatic cancer, non-small cell lung cancer, bladder cancer, breast cancer, and ovarian cancer [7]. We had previously reported that Gem inhibits the viability, growth, and metastasis of OS cells in a murine model [8]. Since then, several clinical trials using Gem have been conducted and recently, additional clinical studies on Gem in combination with docetaxel have been evaluated in various adult sarcomas, including OS [9,10,11,12]. We also reported that Gem strongly induces apoptosis in OS cells in vitro and in vivo [8]; however, several other reports have shown that Gem induces autophagic cell death [13,14,15].

Mammalian target of rapamycin (mTOR) is a protein kinase that is considered to be a key regulator of cellular processes, including cell growth, viability, differentiation, survival, and motility [16]. mTOR has been identified as a key modulator of autophagy, and dysregulation of this pathway has been implicated in a variety of pathological disorders, including cancer [17,18]. The role of autophagy in tumorigenesis is complicated since it appears to play dual roles. On the one hand it promotes cancer progression and drug resistance [19] by allowing tumor cells to escape from apoptosis and promoting their survival [20]. On the other hand, excessive autophagy can cause apoptosis and cell death, thereby inducing autophagic death of drug-resistant tumor cells in malignancies [20]. Rapamycin (Rapa) is an mTOR inhibitor that has shown anti-tumor activity in several human cancers and acts downstream of the phosphoinositide 3-kinase (PI3K) pathway [21,22]. Rapa is prominently known as a strong inducer of autophagy, but it also induces apoptosis [23]. The effects of rapamycin on OS have recently been reported [24,25] and a phase II study exploring the combination of Gem plus mTOR inhibitors in patients with OS in Spain has recently started [26]. However, the efficacy of Rapa in OS treatment has not been fully elucidated.

We hypothesized that treatment with a combination of Gem (which is a potent inducer of apoptosis but weak inducer of autophagy) and Rapa (which is a potent inducer of autophagy but weak inducer of apoptosis) would be a promising approach for the treatment of OS because mechanistically it would prevent tumors from escaping apoptosis by inducing excessive autophagy. We predicted that Rapa would enhance the anti-tumor effects of Gem on OS both in vitro and in vivo by simultaneously inducing apoptosis and autophagy. The current study was conducted to test this hypotheses in OS cells.

## 2. Results

### 2.1. Gem-Rapa Combination Treatment Reduces the Viability, Invasion, and Migration of Osteosarcoma (OS) Cells

**Figure 1:**
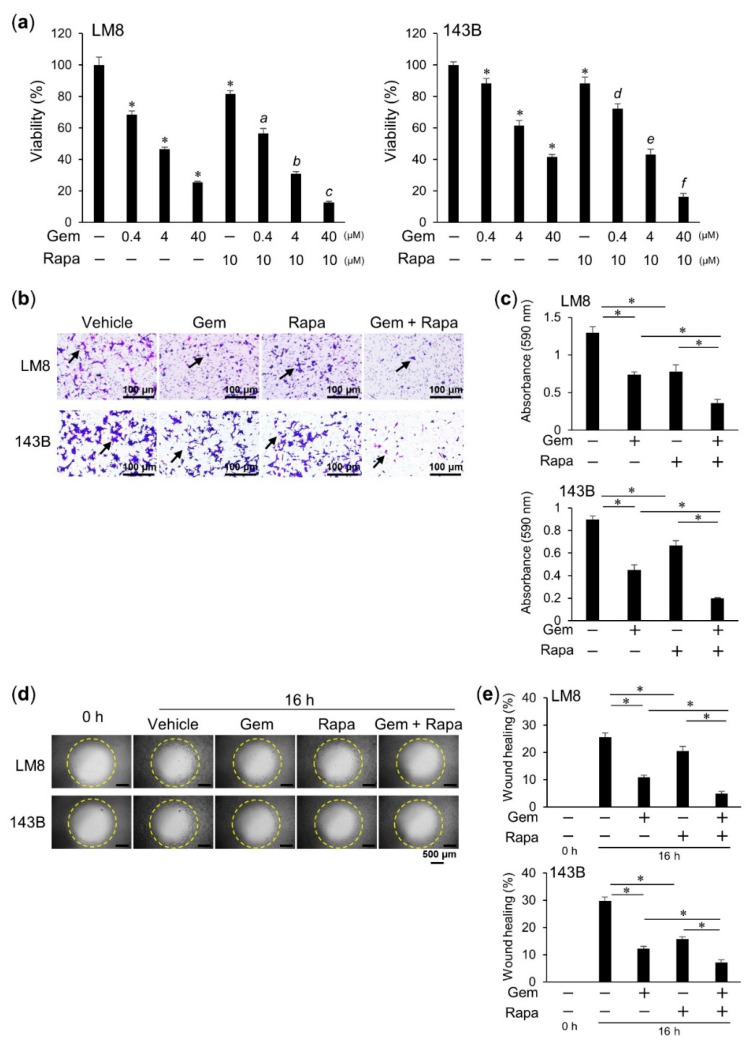
The gemcitabine–rapamycin (Gem–Rapa) combination reduces viability, invasion, and migration of osteosarcoma (OS) cells. (**a**) LM8 and 143B cells were treated with Gem alone or in combination with Rapa and cell viability was measured. Data are shown as the mean ± SD (*n* = 4). * *p* < 0.05 vs. vehicle; a–f, *p* < 0.05 vs. Rapa alone. (**b**) Invasion assays were performed (arrows indicate invaded cells; scale bar, 100 = μm). (**c**) The absorbance of invading cells was quantified. Data represent the mean ± SD (*n* = 3). * *p* < 0.05. (**d**) Cell migration was evaluated. LM8 and 143B cells were treated with Gem alone or in combination with Rapa (scale bar, 500 = μm). (**e**) Pre-migration and post-migration images were analyzed. Data are represented as the mean ± SD (*n* = 3). * *p* < 0.05.

First, we examined the ability of a combination of Gem-Rapa to reduce cell viability. We assessed the individual cell growth inhibitory effects of Rapa and Gem in osteosarcoma cell lines prior to treatment with a combination of these agents. We found that the growth inhibitory effect of Rapa was not dose-dependent as in case of Gem, and that it became strongly inhibitory at 10 μM. Therefore, 10 μM was adopted as the minimum concentration of Rapa that affected osteosarcoma cells (Supplementary Figure S1a). Therefore, OS cells were treated with various concentrations of Gem plus 10 μM Rapa for 72 h and cell viability was analyzed using the water-soluble tetrazolium (WST) dye-based WST-8 cell growth assay. Gem, alone or in combination with Rapa, significantly reduced the viability of OS cell lines LM8, 143B, HOS, and MG63 in a dose-dependent manner (Figure 1a and Supplementary Figure S1b), whereas treatment with the Gem-Rapa combination only modestly decreased the viability of human osteoblast hFOB 1.19 cells (Supplementary Figure S1c). Gem and Rapa individually reduced cell invasion and migration and the Gem–Rapa combination further enhanced these reductions (Figure 1b,e). These results indicate that the Gem–Rapa combination can additively reduce the viability, invasion, and migration of OS cells in vitro.

### 2.2. Gem Induced Apoptosis in OS Cells

**Figure 2:**
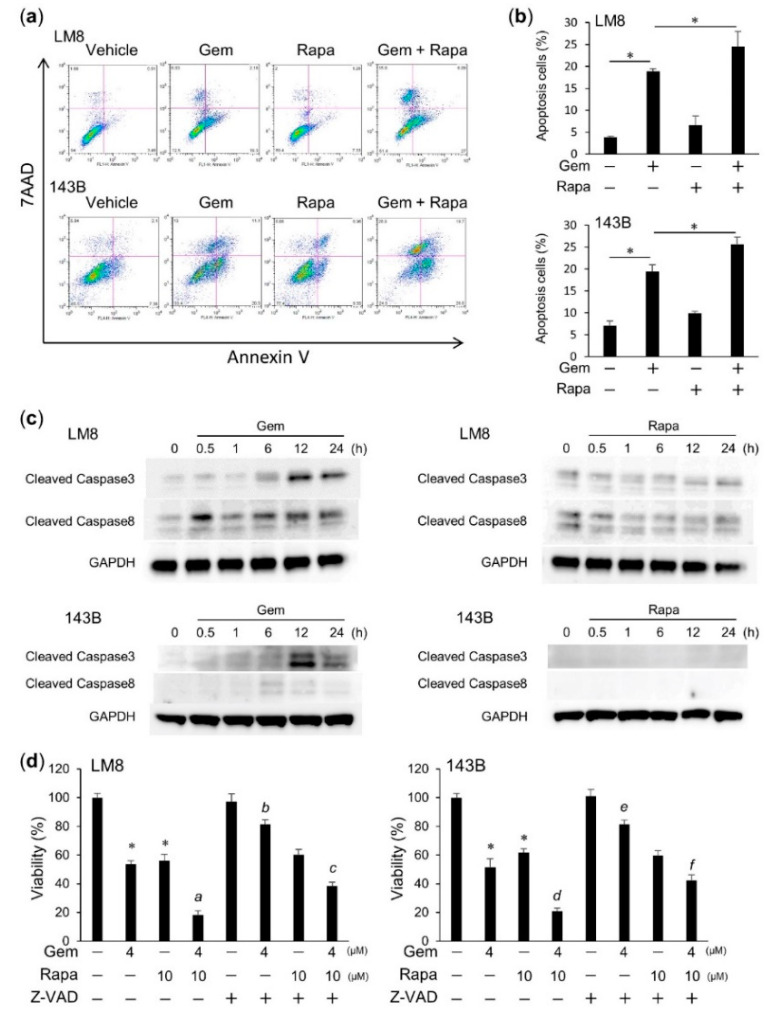
Gem induced apoptosis in osteosarcoma (OS) cells. (**a**) LM8 and 143B cells were treated with Gem alone or in combination with Rapa and cell death assays were performed. (**b**) The rate of cell apoptosis (Annexin V-positive and 7AAD-negative). Data are shown as the mean ± SD (*n* = 3). * *p* < 0.05. (**c**) Caspase-3 and caspase-8 activity in OS cells. LM8, and 143B cells were treated with Gem or Rapa and the levels of cleaved caspase-3 and cleaved caspase-8 were analyzed. See Supplementary Figures S2–S4 for examples of uncropped images and quantification for each antibody. (**d**) LM8 and 143B cells were treated with Gem alone or in combination with Rapa or (benzyloxycarbonyl-Val-Ala-Asp(OMe)-fluoromethylketone (Z-VAD-FMK) (Z-VAD, 10 μM) and cell viability was measured. Data are shown as the mean ± SD (*n* = 4). * *p* < 0.05 vs. vehicle; a, b, d, and e, *p* < 0.05 vs. Gem alone; c and f *p* < 0.05 vs. Gem plus Rapa.

Double staining with fluorescein isothiocyanate (FITC)-conjugated Annexin V and 7-aminoactinomycin D (7-AAD) followed by flow cytometric analyses showed that Gem significantly increased the rates of apoptosis in LM8 and 143B cells (Annexin V-negative and 7-AAD-positive staining). The combination of Gem and Rapa treatment enhanced this increase, although Rapa alone did not increase the apoptosis rate compared with the vehicle (Figure 2a,b). Western blot analysis showed the levels of activated caspase-3 (cleaved caspase-3 (CC3)), activated caspase-8 (cleaved caspase-8 (CC8)) increased following Gem treatment. In contrast, Rapa treatment did not increase the levels of these caspases (Figure 2c and Supplementary Figures S2–S4). To confirm these results, we examined the effect of pharmacological inhibitors specific for apoptosis. The broad-spectrum caspase inhibitor (benzyloxycarbonyl-Val-Ala-Asp(OMe)-fluoromethylketone (Z-VAD-FMK) significantly inhibited cell death in Gem-treated and Rapa-Gem-combination‒treated cells, but not in cells treated with Rapa alone (Figure 2d). These results indicate that Rapa enhances Gem-induced apoptosis, but Rapa alone does not induce apoptosis in OS cells.

### 2.3. Rapa Induced Autophagic Flux in OS Cells

**Figure 3:**
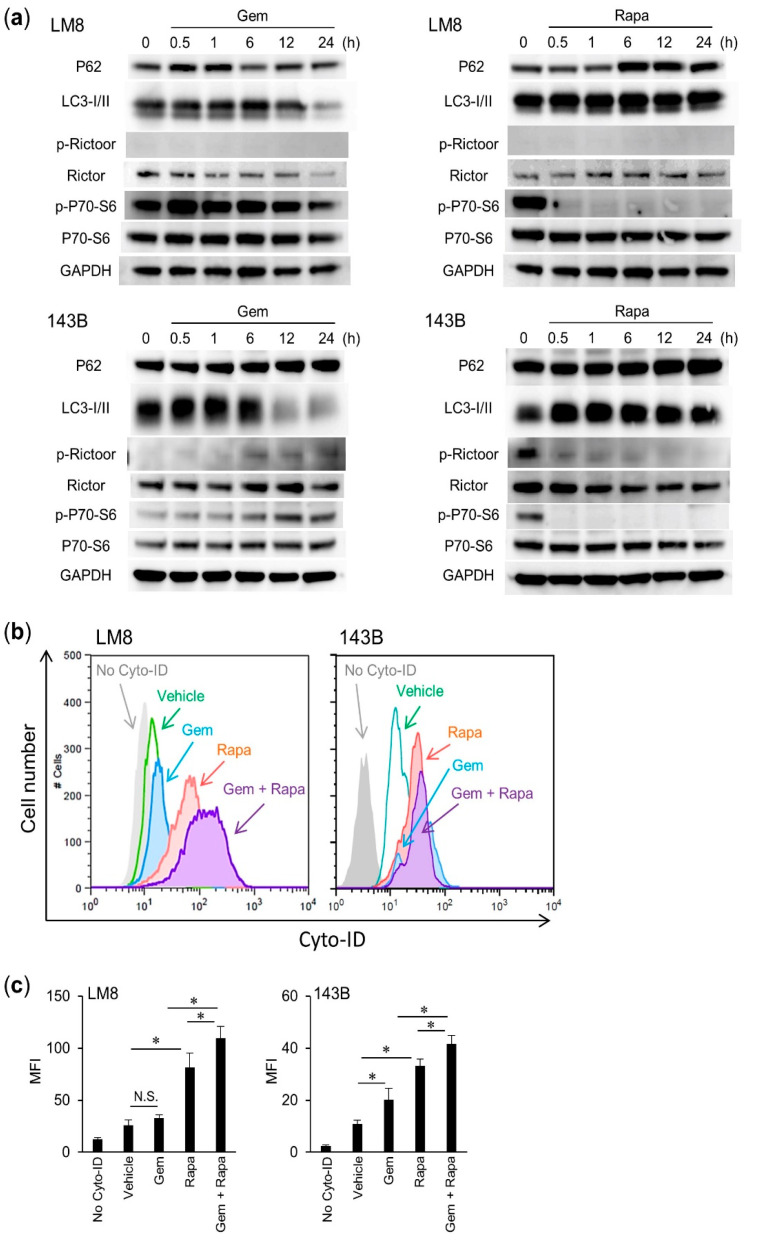
Rapa induced autophagy in OS cells. (**a**) LM8 and 143B cells were treated with Gem or Rapa and the levels of p62, microtubule-associated protein light chain 3 (LC3)-I/II, rapamycin-insensitive companion of mammalian target of rapamycin (RICTOR), and p70-S6 and their phosphorylated forms (as appropriate) were assessed by western blotting. See Supplementary Figures S5–S8 for examples of uncropped images and quantification the bands for each protein. (**b**,**c**) LM8 and 143B cells were loaded with Cyto-ID and treated with Gem alone or in combination with Rapa and analyzed by flow cytometry. Data are shown as the mean ± SD (*n* = 3). * *p* < 0.05. MFI, median fluorescence intensity. N.S., not significant.

We investigated the effect of Gem and Rapa on autophagic flux in OS cells. The mTOR complex (mTORC) plays a pivotal role in regulating autophagy by inhibiting phagophore formation [27,28,29]. To determine whether this complex participates in the promotion of autophagy, we examined the effect of Gem and Rapa on mTORC activation in LM8 and 143B cells by western blotting. The results showed that Rapa increased the levels of p62 and the autophagosomal marker microtubule-associated protein light chain 3 (LC3)-I/II and decreased the phosphorylation of p70-S6 kinase (the substrate of mTOR kinase 1/2) and rapamycin-insensitive companion of mammalian target of rapamycin (RICTOR), which is a component of these two kinases. In contrast, Gem reduced LC3-I/II levels and increased the phosphorylation of p70-S6 and RICTOR (Figure 3a and Supplementary Figures S5–S8). To confirm these results, LM8 and 143B cells were analyzed by flow cytometry after treatment with Cyto-ID, a specific probe for autophagosome formation. Rapa treatment increased the Cyto-ID signal intensity. Moreover, Gem combined with Rapa enhanced this increase. However, quantitatively, there were differences in the median fluorescence intensity (MFI) of Cyto-ID in the two different cell lines. Compared to the background, the MFI baseline was higher in 143B cells than in LM8 cells. Gem alone did not increase the MFI signal in LM8 cells compared with that of the vehicle, but increased the MFI signal in 143B cells (Figure 3b,c). These results indicate that Gem enhances the induction of autophagy by Rapa, but that the induction of autophagy by Gem itself varies depending on the type of OS cell line.

### 2.4. Combined Administration of Gem and Rapa Reduced the Growth and Metastasis of LM8 Subcutaneous Allografts

**Figure 4:**
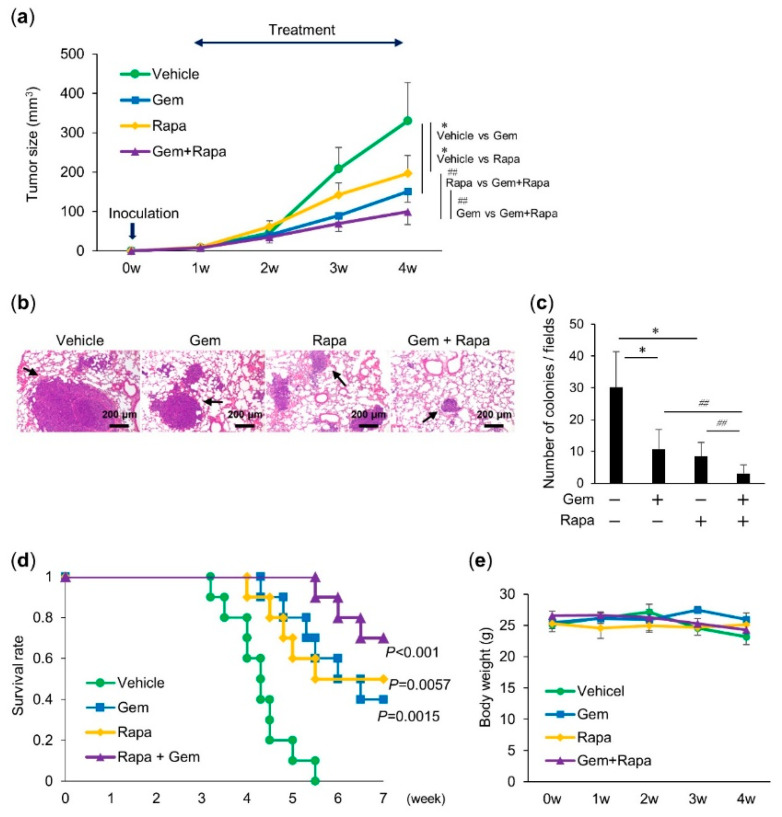
Combined administration of Gem and Rapa reduced the growth and metastasis of LM8 cells in a murine subcutaneous allograft model. (**a**) C3H mice were subcutaneously injected with LM8 cells and treated with Gem, Rapa, or the combination. Tumor sizes were measured weekly. Values represent the mean ± SD (*n* = 10 tumors). * *p* < 0.05 compared with the vehicle. *## p* < 0.05 vs. Gem or Rapa alone. (**b**) Representative micrographs of the lungs (hematoxylin–eosin staining). **Arrows** indicate metastatic nodules; bar = 200 μm. (**c**) Quantitative analysis of metastatic nodules in the lungs. Results are shown as mean ± SD. * *p <* 0.05 compared with the vehicle. *## p <* 0.05 vs. Gem or Rapa alone. (**d**) Survival rate was determined based on a Kaplan–Meier plot (*n* = 10). Data were analyzed using a log-rank test. (**e**) The weights of the mice were measured weekly for 4 weeks. Data are shown as the mean ± SD (*n* = 10). See Supplementary Figure S9 for a schematic flowchart and photos of tumor-bearing mice.

We next determined whether Gem and Rapa would cooperatively suppress the growth and metastasis of OS in vivo. LM8 cells transplanted into the skin of C3H mice induce the development of tumors with high metastatic potential that can spread to the lung. We assessed the anticancer activity of Gem and Rapa individually and in combination using this allograft model (Supplementary Figure S9). Intravenous administration of Gem and Rapa to mice resulted in significant (*p* = 0.017 and *p* = 0.02) reduction in primary tumor size (54.3% and 40.3%, respectively; Figure 4a). A more pronounced anticancer effect was observed in mice treated with the combination of Gem and Rapa; a 69.9% reduction in primary tumor size was observed (Figure 4a). Histological analysis using hematoxylin–eosin (H&E) staining revealed that Gem and Rapa significantly decreased the number of metastatic nodules in the lungs of LM8-inoculated mice. Combinatorial therapy with Gem and Rapa was more effective than monotherapy (Figure 4b,c). The survival rate after treatment with Gem or Rapa was significantly higher than that of the vehicle. Upon combining Gem with Rapa a further increase in the survival rate was observed (Figure 4d). Despite the potent anticancer effect, there was minimal difference in weight between mice in the treated and untreated groups and no obvious abnormalities were observed in mice treated using this protocol (Figure 4e). These results indicate that combinatorial therapy with Gem and Rapa had an improved anticancer effect compared to that of monotherapies in the LM8 subcutaneous allograft model.

### 2.5. Combined Administration of Gem and Rapa Reduced the Growth and Metastasis of 143B Intramedullary Xenografts

**Figure 5:**
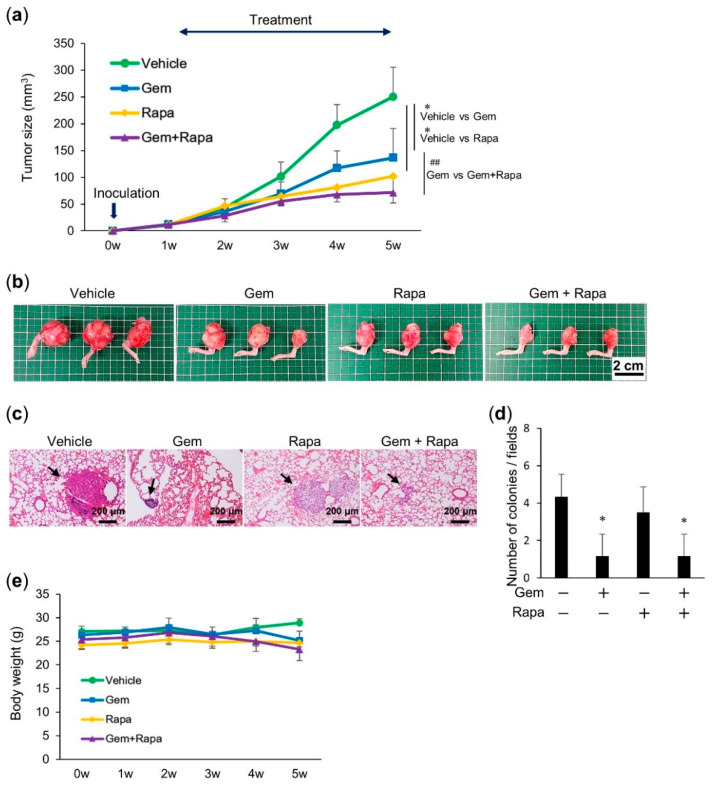
Combined administration of Gem and Rapa reduced the growth and metastasis of 143B cells in a murine intramedullary xenograft model. (**a**) BALB/c nu/nu mice were transplanted with 143B cells and treated with Gem, Rapa, or the combination. Tumor sizes in the mice were measured weekly. Values represent the mean ± SD (*n* = 10). * *p* < 0.05 compared with the vehicle. *## p* < 0.05 vs. Gem alone. (**b**) Representative photos of primary tumors at 5 weeks after the transplantation of 143B cells. Bar = 2 cm. At the same time, the lungs were removed and metastatic nodules were counted. (**c**) Representative micrographs of the lungs (hematoxylin–eosin staining). Arrows indicate metastatic nodules; bar = 200 μm. (**d**) Quantitative analysis of metastatic nodules in the lungs. Results are shown as mean ± SD. * *p <* 0.05 compared with the vehicle. (**e**) The weights of the mice were measured weekly for 5 weeks. Data are the mean ± SD (*n* = 10). See Supplementary Figure S9 for a schematic flowchart.

A second murine xenograft model was used to verify whether Gem and Rapa cooperatively suppressed the growth and metastasis of OS cells. BALB/c nu/nu mice injected with human 143B cells using intramedullary transplantation in the tibias, develop tumors with metastatic potential that can spread to the lungs. We used this xenograft model to further assess the anticancer activity of Gem and Rapa alone or in combination. Treatment of the xenografted mice via intravenous administration of Gem and Rapa resulted in significant (*p* = 0.026 and *p* = 0.005) reductions in primary tumor size (45.4% and 59.2%, respectively; Figure 5a,b). Once again, a more pronounced anticancer effect was observed in mice treated with the combination of Gem and Rapa, there being a 71.4% reduction in primary tumor size (Figure 5a,b). The combination of Gem and Rapa had a significant tumor-reducing effect compared with Gem alone, but was not significantly different from that of Rapa alone. This result is different from that obtained in the LM8 subcutaneous allograft model. One possible explanation for this might be that the two cell lines have different autophagic fluxes (see Figure 3). Histological analysis using H&E staining revealed that Gem either alone or in combination with Rapa significantly decreased the number of metastatic nodules in the lungs of 143B-xenografted mice (Figure 5c,d). As with the BALB/c nu/nu xenograft model, there was minimal difference in weight between mice in the treated and untreated groups and again no obvious abnormalities were observed in any of the mice treated using this protocol (Figure 5e). These results indicate that the combined administration of Gem and Rapa also exhibited an improved anticancer effect, compared monotherapies in the 143B intramedullary xenograft model.

### 2.6. Combined Administration of Gem and Rapa Reduced the Ki-67 Labeling Index

**Figure 6:**
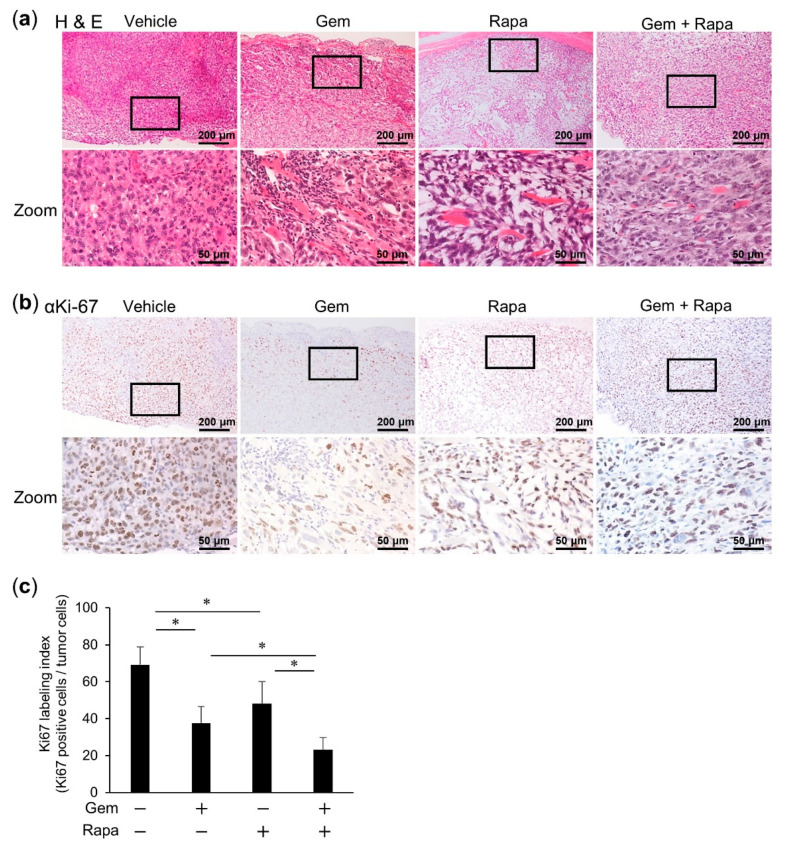

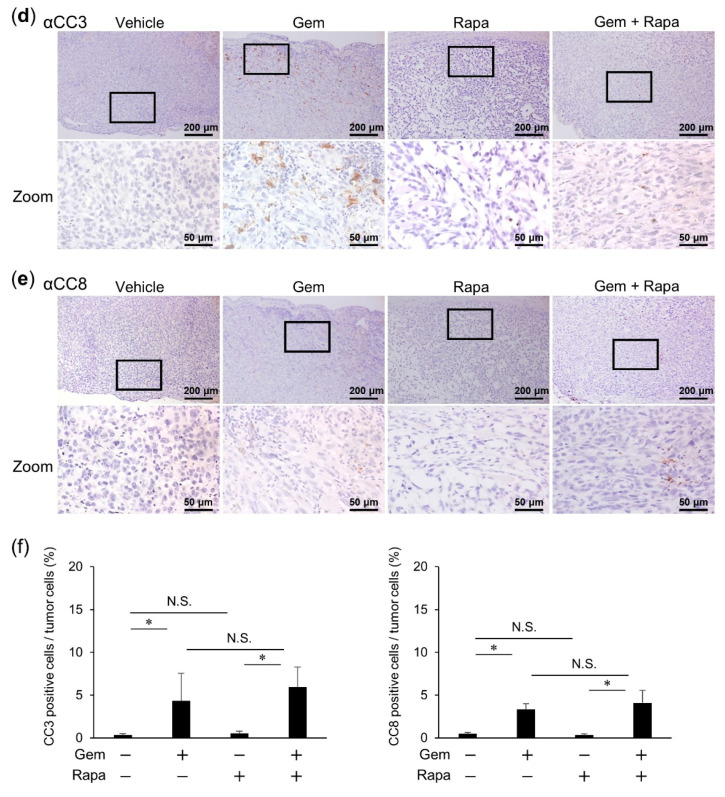
Combined administration of Gem and Rapa reduced the Ki-67 labeling index. Primary tumors were resected at day 35 after transplantation of 143B cells. (**a**–**c**) Hematoxylin–eosin (H&E) staining and immunohistochemical staining were performed using primary antibodies against Ki-67, and Ki-67-positive cells were counted and determine the Ki67 labeling index. (**d**–**f**) Immunohistochemical staining was performed to measure the levels of cleaved caspase-3 (αCC3) and cleaved caspase-8 (αCC8), followed by quantification. Bar = 200 μm in the upper panels. Bar = 50 μm in the lower panels. Data are shown as the mean ± SD (*n* = 5). * *p* < 0.05. N.S., not significant.

The tumor proliferation index marker Ki-67 is intimately associated with tumor-cell proliferation [30,31]. The Ki-67 labeling indexes of primary tumors resected on day 35 post-transplantation of 143B cells were significantly lower for both the Gem- and Rapa-treated groups compared to those of the vehicle group. Combined treatment with Gem and Rapa resulted in a significant reduction in the proliferation index compared to either of the monotherapies (Figure 6a–c). The CC3 and CC8 levels are shown in Figure 6d–f, respectively, for Gem treatment alone and in combination with Rapa. These results were in agreement with those obtained in the in vitro experiments.

### 2.7. Combined Administration of Gem and Rapa Induced the Autophagic Flux in Vivo

**Figure 7:**
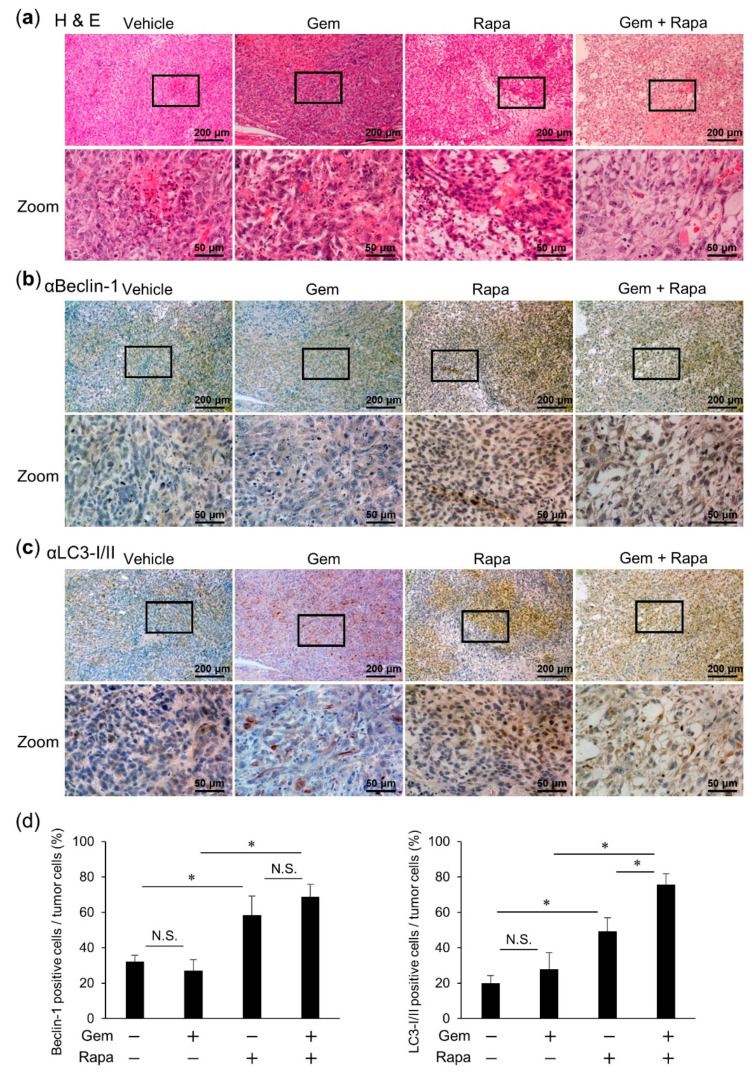
Combined administration of Gem and Rapa induced the autophagic flux in vivo. (**a**–**d**) Primary tumors were resected at day 28 after transplantation of LM8 cells. Hematoxylin-eosin (H & E) staining and immunohistochemical (IHC) staining were performed using primary antibodies against Beclin-1 and LC3-I/II, followed by quantification. Bar = 200 μm in the upper panels. Bar = 50 μm in the lower panels. Data are shown as the mean ± SD (*n* = 5). * *p* < 0.05. N.S., not significant.

The impact of Gem and Rapa on autophagy in vivo was determined by immunohistochemical (IHC) against Beclin-1 and LC3-I/II. The expression of Beclin-1 and LC3-I//II was higher in the Rapa treatment group compared to that in the Gem-treated and vehicle groups. Combined treatment with Gem and Rapa enhanced Beclin-1 and LC3-I//II expression compared to that in the Rapa only treatment group (Figure 7a–d). These results are consistent with those of the in vitro experiments.

### 2.8. Combined Administration of Gem and Rapa Reduced Angiogenesis and Vascular Endothelial Growth Factor (VEGF) Production

**Figure 8:**
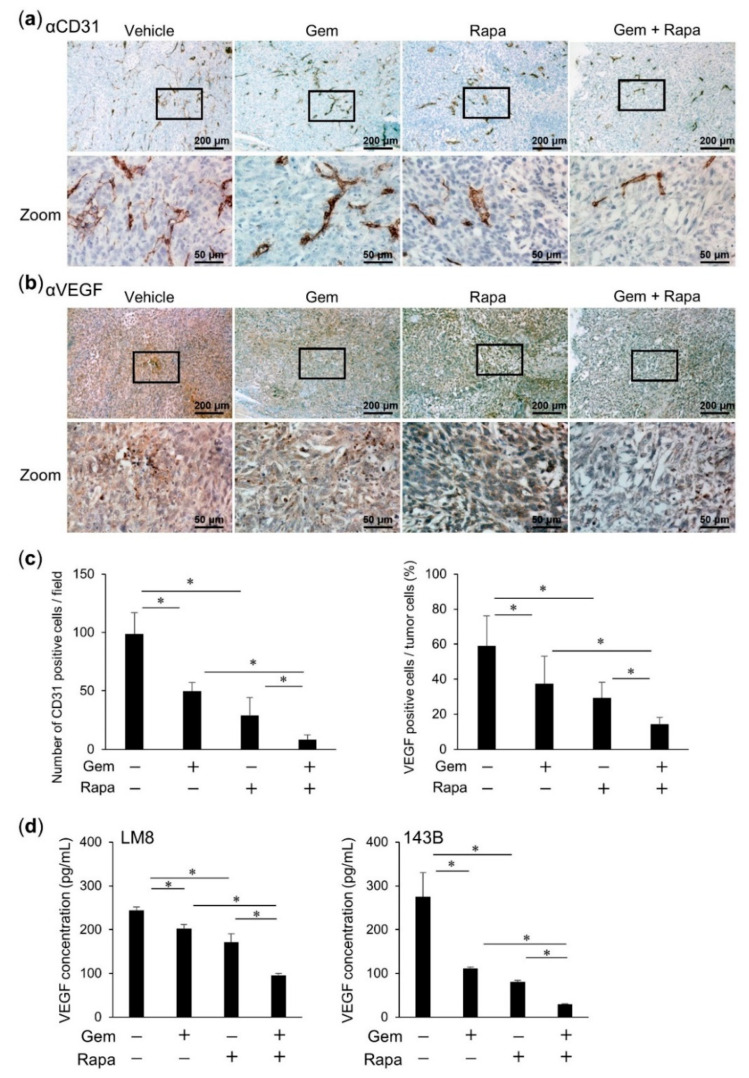
Combined administration of Gem and Rapa reduced angiogenesis and Vascular Endothelial Growth Factor (VEGF) production. Primary tumors were resected at day 28 post-transplantation of LM8 cells. Angiogenesis was evaluated by IHC against CD31 (**a**) and VEGF (**b**,**c**) Cells positive for CD31 and VEGF were counted and quantified. Bar = 200 μm in the upper panels. Bar = 50 μm in the lower panels. (**d**) LM8 and 143B cells were treated with Gem alone or in combination with Rapa. The levels of VEGF in the supernatant were determined using ELISA. Results are shown as the mean ± SD. * *p* < 0.05.

Finally, we evaluated angiogenesis in primary OS tumors. Vascular endothelial cells were identified by IHC against CD31. The expression of CD31 was lower in the Gem and Rapa treatment groups compared to that in the vehicle group. A more pronounced reduction in CD31 expression was observed in the tumors of mice treated with the combination of both Gem and Rapa (Figure 8a,c). We also investigated Vascular Endothelial Growth Factor (VEGF) expression levels in vivo using IHC and in vitro using ELISA. Gem and Rapa both significantly decreased VEGF production both in vivo and in vitro. Moreover, the combination of both Gem and Rapa enhanced these in vivo and in vitro reductions compared with the single administration of either agent (Figure 8b–d). These results suggest that the combined administration of Gem and Rapa suppresses angiogenesis to a greater extent than either treatment alone.

## 3. Discussion

Most OSs are treated with multiple chemotherapy agents for approximately three months before surgery and then again for up to a year after surgery. Although chemotherapy based on methotrexate, doxorubicin, cisplatin, and ifosfamide has improved the prognosis of OS, the 5-year survival rate has changed little in the last 20 years [32,33]. The complications and fatal toxicities caused by these agents are also major problems that need to be solved. Furthermore, the treatment of metastatic or recurrent OS after standard chemotherapy remains an issue. Therefore, there have been several clinical trials to evaluate potential second-line and further lines of therapy [34,35,36]. Current research in patients with metastatic OS focuses on apoptosis-mediated surface antigens and the mTOR pathway [26,37]. The efficacy of Gem has been shown in both children and adolescents with refractory OS [11,12]. A phase II study on the efficacy of mTOR inhibitors (ridaforolimus and sirolimus) at treating sarcomas has also been reported [38,39]. Additionally, a phase I study on the treatment of advanced soft tissue sarcoma and a phase II study on the treatment of OS using a combination of Gem plus mTOR inhibitors are underway [26,40]. In the current study, we showed that combinatorial treatment with Gem and Rapa reduced cell viability, cell invasion, and migration—in human and murine OS cell lines—induced apoptotic and autophagic cell death, and reduced OS tumor growth and metastasis in vivo. Notably, this combination of Gem and Rapa showed minimal cytotoxicity in osteoblasts. Furthermore, treatment with these agents did not result in any significant adverse effects, including weight loss in vivo.

In this study, we were able to induce apoptosis in OS cells. We found that treatment of OS cells with Gem significantly reduced the viability in a dose-dependent manner, increased the number of apoptotic cells, and activated caspase-3 and caspase-8. IHC staining also showed that Gem induced CC3 and CC8 expression in vivo. These observations are consistent with those presented in our previous report [8] and those of others [7,15]. Gem induces apoptosis more potently than other basic agents, such as methotrexate, doxorubicin, cisplatin, and ifosfamide, and may therefore be a key drug for treating OS through the induction of apoptosis. In contrast, previous reports have shown that Rapa either suppresses [41,42] or induces [43,44] apoptosis, depending on the cell type and environment [45]. In the current study, we found that the combination of Gem and Rapa enhanced the induction of apoptosis to a greater extent than Gem treatment alone, whereas Rapa alone failed to increase the rate of apoptosis or to induce an increase in CC3 and CC8 expression. 

Rapa is known to promote autophagy by blocking the mTOR pathway [46,47]. A correlation has been identified between mTOR overexpression and oncological progression and worse prognosis in patients with OS [48]. In addition, mTORC is known to play a pivotal role in regulating autophagy by inhibiting phagophore formation [27,28,29]. Accordingly, we investigated the effect of the combination of Gem and Rapa on autophagic flux. Rapa reduced cell viability and induced autophagy in OS cells by increasing autophagosome formation and suppressing the mTORC pathway. The role of autophagy in tumorigenesis is complicated as it appears to play dual roles. On the one hand it promotes cancer progression and drug resistance [19] by allowing tumor cells to escape from apoptosis and promoting their survival [20]. On the other hand, excessive autophagy can cause apoptosis and cell death, thereby inducing autophagic death of drug-resistant tumor cells in malignancies [20]. Treatment with Z-VAD did not ameliorate the reduction in cell viability induced upon treatment with Rapa alone. Based on these data we concluded that Rapa alone does not induce apoptosis in OS cells at the concentration tested. However, it cannot be ruled out that other concentrations of Rapa might affect apoptosis. When cells were treated with a combination of the Gem and Rapa, Rapa did not directly induce apoptosis, however the excessive autophagy induced by Rapa prevents escape from the Gem-induced apoptosis. As a result, we conclude that the number of apoptotic cells is increased upon treatment with a combination of Gem and Rapa. In a tumor-bearing murine model, Rapa also increased autophagic flux compared with vehicle treatment. In contrast to the results of the apoptosis analysis, Gem alone did not increase the autophagic flux, either in vitro or in vivo, but the combination of Gem and Rapa did enhance Rapa-induced autophagy. These results indicate that the effects of Gem and Rapa were complementary, one accentuating what the other induced—with respect to apoptosis and autophagy—without suppressing each other.

Angiogenesis refers to the growth of new vessels initiating from the preexisting vasculature. Angiogenesis in the tumor environment results in a heterogeneous structure and permeable blood vessels [49]. VEGF and its receptor are known to play a prominent role in tumor angiogenesis [50]. Recently, circulating VEGF in blood has been evaluated as a predictive biomarker during antiangiogenic therapy in gastric [51], ovarian [52], and colorectal [53] cancers. In the current study, we showed that Gem and Rapa significantly decreased angiogenesis and VEGF production in primary tumors from murine OS transplant models. Moreover, the combination of Gem and Rapa enhanced these reductions in angiogenesis and VEGF production compared with individual monotherapies. These reductions in VEGF production and angiogenesis in response to this combination therapy may have contributed to the suppression of metastasis and improvement of prognosis in OS.

This study had some limitations. First, although we showed that combinatorial treatment with Gem and Rapa reduced cell invasion, cell migration, VEGF production, and angiogenesis, we did not investigate whether these outcomes directly contributed to the suppression of metastasis and the improvement in survival. Second, we demonstrated a suppressive effect on OS via apoptotic and autophagic cell death in vitro and in vivo; however, it is possible that other mechanisms may also regulate tumor growth and metastasis of OS, such as necroptosis, pyroptosis, ferroptosis, or mitophagy. Third, the detailed mechanism of the additive effects as observed in case of the combination therapy—with Gem inducing apoptosis and Rapa inducing autophagy—remains unclear. Collapse of the mitochondrial network or structure may be related to this additive effect. Further study is therefore needed to examine the specific mechanisms in OS cells in response to the combination treatment of Gem and Rapa.

For the clinical use of Gem in chemotherapy for cancer, its combination with another drug, rather than use as monotherapy, is more promising and practical. In this regard, it is notable that Gem and Rapa act as mutual adjuvants to one another in suppressing tumor growth and metastasis. These findings indicate that the combined use of Gem and Rapa may significantly reduce the effective dose levels, thereby limiting or preventing adverse effects. The combination therapy of Gem with Rapa shows promise as a novel strategy for the treatment of OS.

## 4. Materials and Methods

### 4.1. Cell Culture and Reagents

The murine OS cell line LM8 (RCB1450), and human OS cell lines 143B (RCB0701), HOS (RCB0992), and MG63 (RCB1890) were purchased from Riken Cell Bank (Tsukuba, Japan). The human osteoblast cell line hFOB 1.19 (ATCC CRL-11372) was purchased from American Type Culture Collection (Manassas, VA, USA). Cells were cultured in growth media consisting of Dulbecco’s Modified Eagle’s Medium (DMEM) supplemented with 4.5 g/L glucose (Invitrogen, Carlsbad, CA, USA), 10% fetal bovine serum (FBS), 100 U/mL penicillin, and 100 μg/mL streptomycin at 37 °C in a humidified atmosphere containing 5% CO_2_. All cell cultures were tested for mycoplasma contamination using a MycoAlert Mycoplasma Detection Kit (Lonza, Ann Arbor, MI, USA). Gem and Rapa were purchased from Sigma-Aldrich (St. Louis, MO, USA). The pan-caspase inhibitor Z-VAD-FMK was purchased from Merck Millipore (Darmstadt, Germany).

### 4.2. Cell Viability Assay

Cell viability was measured using a Cell Counting Kit-8 (WST-8; Dojindo Molecular Technologies, Inc., Kumamoto, Japan) following the manufacturer’s instructions. Cells were seeded into 96-well plates at 5 × 10^3^ cells/well and cultured in DMEM containing 10% fetal calf serum (FCS) and different concentrations of Gem (0.4, 4, and 40 µM) with or without Rapa (10 μM) for 72 h at 37 °C. Cell counting reagent (10 μL) was then added and the cells incubated for another 2 h. The culture absorbance was measured at 450 nm using an SH-1100R microplate reader (Corona Electric Co., Ibaraki, Japan).

### 4.3. Cell Invasion Assay

OS cells were seeded onto Chemotaxicell filters with a pore size of 5 μm (Kurabo, Tokyo, Japan) that had been coated with 300 µg/mL Atelocollagen (KOKEN CO., Tokyo, Japan). A schematic of the invasion assay setup is depicted in Supplementary Figure S1c. The upper and lower chambers were separated using filters with 5-µm pores. In the lower chambers of the 24-well plate, 1 mL of DMEM containing Gem (4 μM) alone or in combination with Rapa (10 μM) was added. Next, 1 × 10^5^ LM8 or 143B cells in 500 µL DMEM were added to the upper chambers of the wells. After incubation for 24 h at 37 °C, the membranes were removed and washed with phosphate-buffered saline. Non-invaded cells on the upper surface of the membrane were removed using a cotton swab. The remaining cells on the membranes were fixed in 4% paraformaldehyde for 5 min, stained with 1% crystal violet solution (Sigma-Aldrich), and then dissolved in 99% ethanol before quantification by absorbance at 590 nm using the SH-1100R microplate reader.

### 4.4. Cell Migration Assay

Cell migration was evaluated using an Oris Cell Migration Assay kit (Platypus Technologies, Madison, WI, USA), according to the manufacturer’s instructions. Cells (5 × 10^4^) were seeded into each well of the 96-well plates. After 1 h pretreatment with 1% FBS/DMEM containing Gem (4 μM) alone or in combination with Rapa (10 μM), the stoppers were removed to allow the cells to migrate into the detection zone. The cells were then incubated for 16 h, fixed with 4% paraformaldehyde for 5 min, stained with 1% crystal violet, and photographed. The pre-migration and post-migration images were analyzed using ImageJ v. 1.52a (Wayne Rasband, National Institutes of Health, Washington, DC, USA).

### 4.5. Cell Death Assay

Cells (5 × 10^5^) were cultured in 6-well plates for 12 h and then exposed to 4 µM of Gem alone or in combination with 10 µM of Rapa for 24 h. To evaluate apoptotic cell death, the cells were retrieved using Versene (GIBCO^®^, Life Technologies, Carlsbad, CA, USA) and incubated with Annexin V and 7AAD (BD Biosciences) for 15 min. Data were collected using a FACSCalibur flow cytometer (BD Biosciences, Franklin Lakes, NJ, USA). Annexin V-positive and 7AAD-negative cells were defined as early apoptotic cells. Data obtained were analyzed using FlowJo software (TreeStar Inc., Palo Alto, CA, USA). Experiments were performed in triplicate.

### 4.6. Western Blotting Analysis

LM8 and 143B cells were collected and cell lysates were prepared using a CelLytic MT cell lysis reagent (Sigma-Aldrich, St. Louis, MI, USA) according to the manufacturer’s instructions. Western blotting was then performed to measure protein expression. Equal amounts of protein from each sample were analyzed as previously described [54], by immunoblotting with primary antibodies against cleaved caspase-3 (Asp175) (5A1E) (#9664, 1:1000), cleaved caspase-8 (Asp387) (D5B2) (#8592, 1:1000), phospho-P70-S6 (Thr389) (108D2) (#9234, 1:1000), P70-S6 (49D7) (#2708, 1:1000), phospho-RICTOR (Thr1135) (D30A3) (#3806, 1:1000), RICTOR (53A2) (#2114, 1:1000), LC3-I/II (D3U4C) (#12741, 1:1000), and GAPDH (D16H11) (#5174, 1:1000) obtained from Cell Signaling Technology (Danvers, MA, USA); and p62 (SQSTM1) (PM045, 1:1000) obtained from MBL International Corporate (MBLI) (Woburn, MA, USA), were used. Images were captured using a LAS-4000 camera system from Fujifilm (Tokyo, Japan) and quantified using ImageJ 1.52a (Wayne Rasband, National Institutes of Health, Bethesda, MD, USA).

### 4.7. Autophagy Assay

Cells (5 × 10^5^) were cultured in 6-well plates for 12 h and then exposed to 4 µM Gem, alone or in combination with 10 µM Rapa, for 48 h. The cells were then stained and analyzed using a Cyto-ID Autophagy Detection Kit obtained from Enzo Life Sciences, Inc. (Farmingdale, NY, USA), according to the manufacturer’s instructions. Data were collected using the FACSCalibur flow cytometer and analyzed using FlowJo software. Experiments were performed in triplicate.

### 4.8. Animals

All experiments involving the use of mice were conducted according to the Guidelines for Proper Conduct of Animal Experiments, Science Council of Japan. Protocols were approved by the Animal Care and Use Committee (No. 17-11), University of Yamanashi. Male C3H/HeJJcl mice and BALB/cAJcl-nu/nu mice were purchased from CLEA Japan, Inc. (Tokyo, Japan). The mice were housed at 22–24 °C under a 12-h light/dark cycle and were fed standard mouse chow. Water was available ad libitum.

### 4.9. Tumor Growth and Metastasis

The ability of Gem or Rapa to reduce tumor growth in vivo was evaluated using allograft transplants of LM8 or 143B cells in mice. Male C3H/HeJJcl or BALB/cAJcl-nu/nu mice (8 weeks of age) were administered general anesthesia with isoflurane (ISOFLU; Abbott Laboratories, North Chicago, IL, USA) and oxygen. LM8 cells (2 × 10^6^ cells/mouse) in 100 µL DMEM were injected subcutaneously into the backs of the CH3 mice on day 0. Alternatively, 143B cells (1 × 10^6^ cells/mouse) in 50 µL of DMEM were injected intramedullary into the right tibia of the BALB/cAJcl-nu/nu mice on day 0. At day 7 post-transplant, 200 μL of Gem (50 mg/kg) two times per week and Rapa (50 mg/kg) every other day alone or in combination were administered intravenously to ten mice in each group. The mice were weighed and the primary tumors were measured weekly. A schematic diagram of the tumor growth and metastasis experiments is shown in Supplementary Figure S9. Tumors and lungs harvested from mice were fixed in 10% formalin neutral buffer solution for 3 d at 20–25 °C. Tumor and lung specimens were then paraffin-embedded and consecutive 5-μm sections were stained with H&E (Merck, Darmstadt, Germany).

### 4.10. Immunohistochemical (IHC) Staining

IHC staining was performed on tumor samples resected day 35 post-inoculation of 143B cells or day 28 post-inoculation of LM8 cells, as previously described [55]. IHC staining was performed using primary antibodies against cleaved caspase-3 (Asp175) (5A1E) (#9664, 1:2000), cleaved caspase-8 (Asp387) (D5B2) (#8592, 1:1000), Beclin-1 (D40C5) (#3495, 1:400), LC3-I/II (D3U4C) (#12741, 1:2000), CD31(#77699, 1:100) each purchased from Cell Signaling Technology (Danvers, MA, USA), Ki-67 (SP6) (ab16667, 1:100; Abcam, Cambridge, UK), and VEGF (A-20) (sc152, 1:50; Santa Cruz Biotechnology, Inc. Dallas, TX, USA). The Dako Liquid DAB + Substrate Chromogen System (Glostrup, Denmark) was used as substrate-chromogen according to the manufacturer’s specifications. The immunostained specimens were counterstained with hematoxylin.

### 4.11. Statistical Analysis

Data are presented as the mean ± standard deviation (SD) and were analyzed by a one-way analysis of variance followed by Tukey’s post hoc test using add-in software (Excel Toukei Version 4.10; Social Survey Research Information Co., Tokyo, Japan) with Excel 2016 for Windows (SSRI, Tokyo, Japan). For some experiments, significance was determined using a Student’s *t*-test after an F-test. *p* < 0.05 was considered statistically significant.

## 5. Conclusions

In this study, we demonstrated that the administration of a combination of Gem and Rapa exhibited potent anti-tumor activity against OS both in vitro and in vivo compared to either treatment alone. This combination treatment induced cell death by apoptosis and autophagy with the two agents accentuating each other’s activity. In addition, the combined administration of Gem and Rapa resulted in reduced cell invasion, cell migration, and vascular endothelial growth factor production. Moreover, the combination of these agents suppressed tumor growth, angiogenesis, and lung metastasis and prolonged the overall survival of OS in allograft and xenograft murine models with minimal adverse effects. Therefore, combination therapy with Gem and Rapa may be a safe and effective approach for treating apoptosis-resistant OS.

## Supplementary Materials

The following are available online at https://www.mdpi.com/2072-6694/12/11/3097/s1, Figure S1: (a–c) Cell viability of osteosarcoma (OS) cells and human osteoblastic cells. (d) Schematic of the cell invasion assay methodology, Figure S2 and S3: Uncropped Western Blot images, Figure S4: Quantification of cleaved caspase 3, cleaved caspase 8, and GAPDH bands in western blots depicted in Figure 2d. (a) LM8 cells. (b) 143B cells, Figure S5: and S6: Uncropped Western Blot images, Figure S7: Quantification of western blot bands corresponding to p62, LC3-I/II, p-RICTOR, RICTOR, p-P70-S6, P70-S6, and GAPDH in LM8 cell lysates depicted in Figure 3a, Figure S8: Quantification of western blot bands of p62, LC3-I/II, p-RICTOR, RICTOR, p-P70-S6, P70-S6, and GAPDH in 143B cell lysates depicted in Figure 3a, Figure S9: (a) Schematic diagram showing the experimental design for Gem and Rapa treatment of mice with allograft or xenograft osteosarcoma cell transplants. (b) Photos of tumor-bearing mice from Figure 4.
