# Supplementary material for: Gemcitabine and Rapamycin Exhibit Additive Effect against Osteosarcoma by Targeting Autophagy and Apoptosis
Source: Cancers (Basel). 2020 Oct 23;12(11):3097. doi: 10.3390/cancers12113097 (PMC7690839; doi:10.3390/cancers12113097)
Supplement: Supplementary file 1 [file cancers-12-03097-s001.pdf]

Supplementary Materials: Gemcitabine and Rapamycin Exhibit Additive Effect Against Osteosarcoma by Targeting Autophagy and Apoptosis

Takashi Ando, Jiro Ichikawa, Taro Fujimaki, Naofumi Taniguchi, Yoshihiro Takayama and Hirotaka Haro

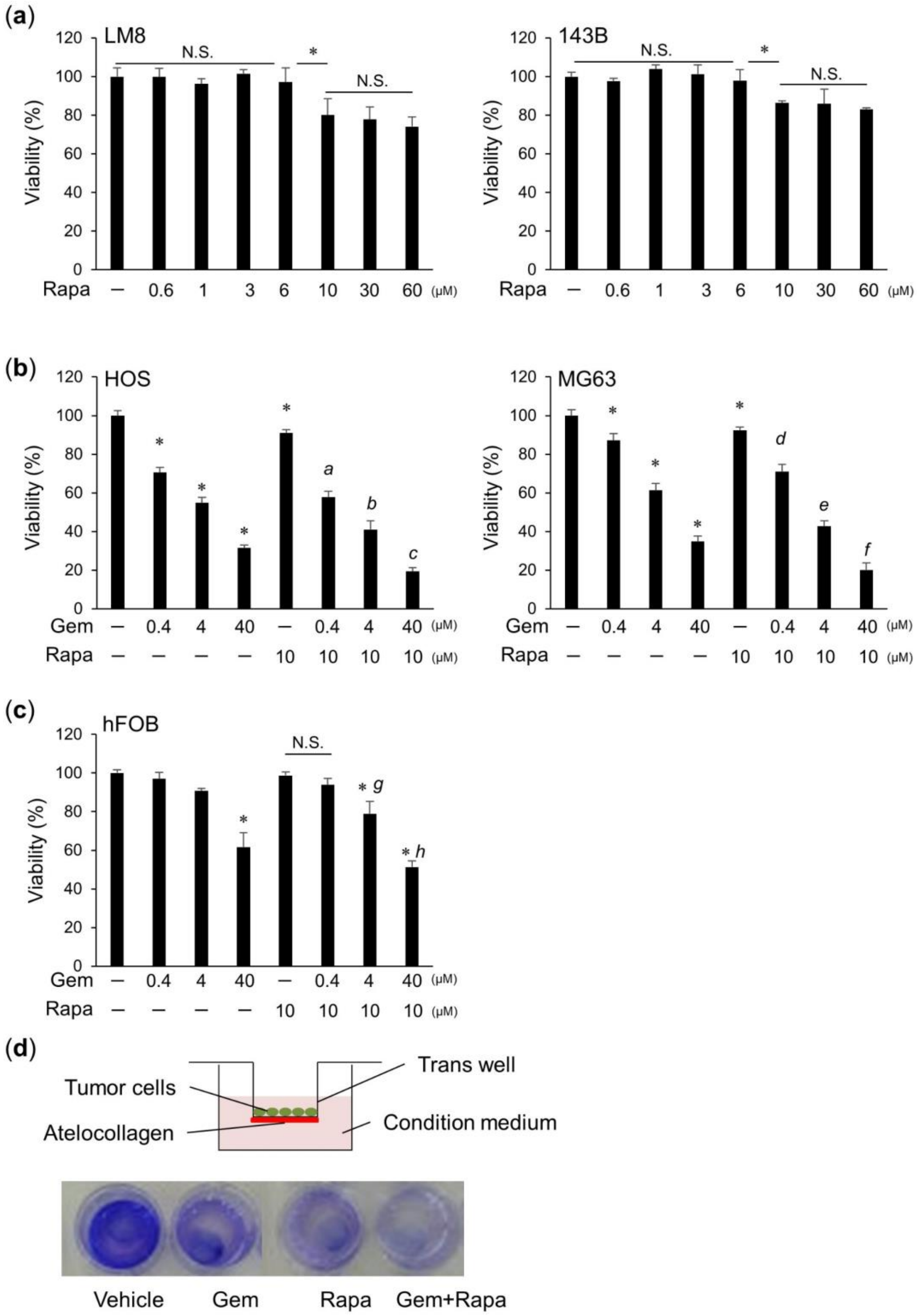

Figure S1. (a) LM8 and 143B OS cell lines were treated with Rapa and cell viability was measured using WST-8 assays. (b) HOS and MG63 OS cells and (c) hFOB osteoblast cells were treated with Gem alone or in combination with Rapa and cell viability was measured using WST-8 assays. Data are shown as the mean ± SD (n = 4). *P < 0.05 vs. vehicle; a–f, P < 0.05 vs. Rapa alone. N.S., not significant. (d) Schematic of the cell invasion assay methodology.

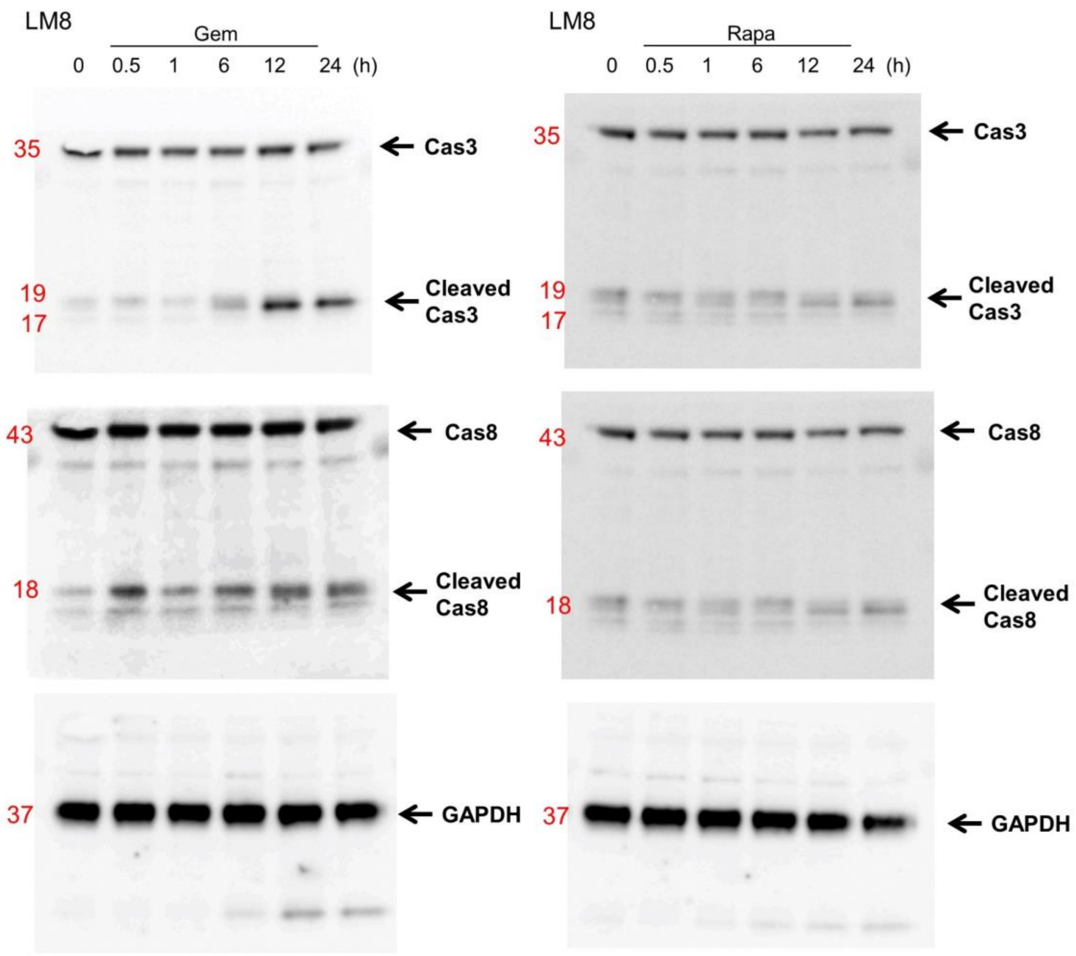

Figure S2. Example uncropped western blots depicting the expression of cleaved caspase 3, cleaved caspase 8, and GAPDH in LM8 cell lysates. These blots complement the cropped blots shown in Figure 2c.

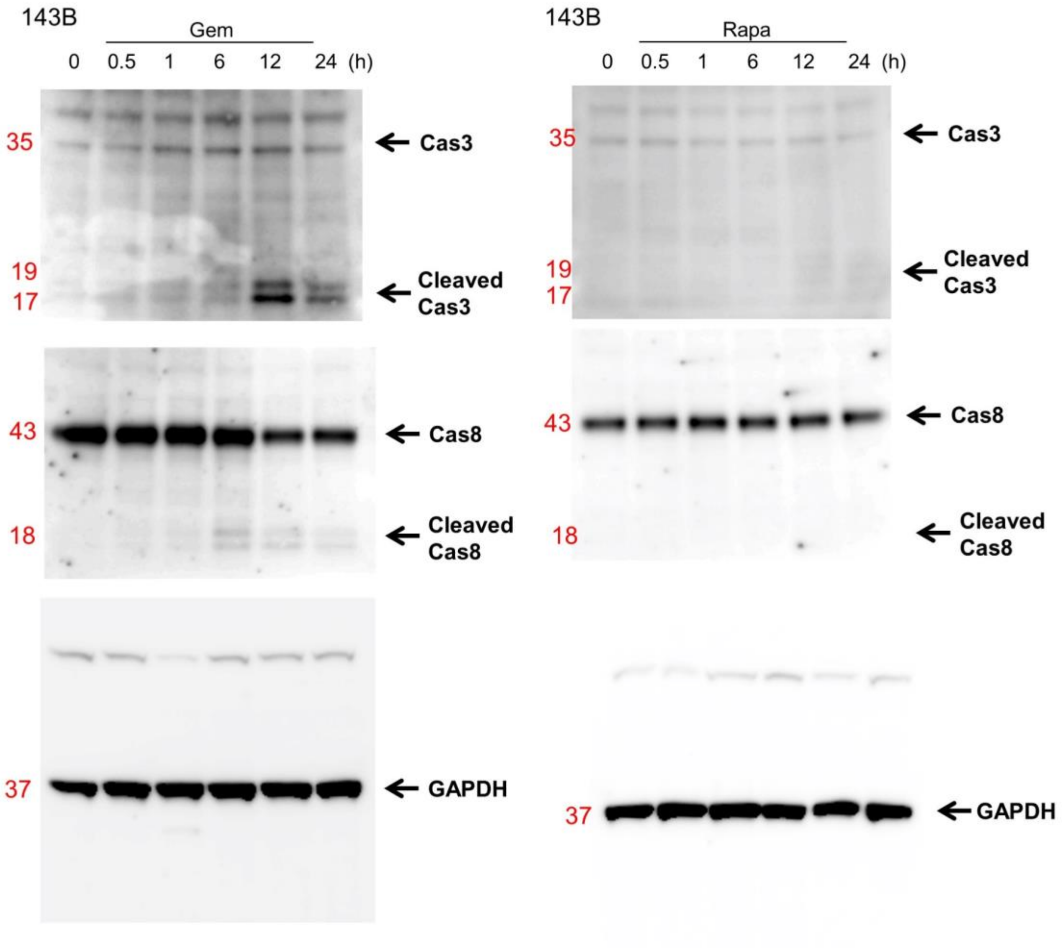

16 **Figure S3.** Example uncropped western blots depicting the expression of cleaved caspase 3, cleaved
17 caspase 8, and GAPDH in 143B cell lysates. These blots complement the cropped blots shown in
18 Figure 2c.

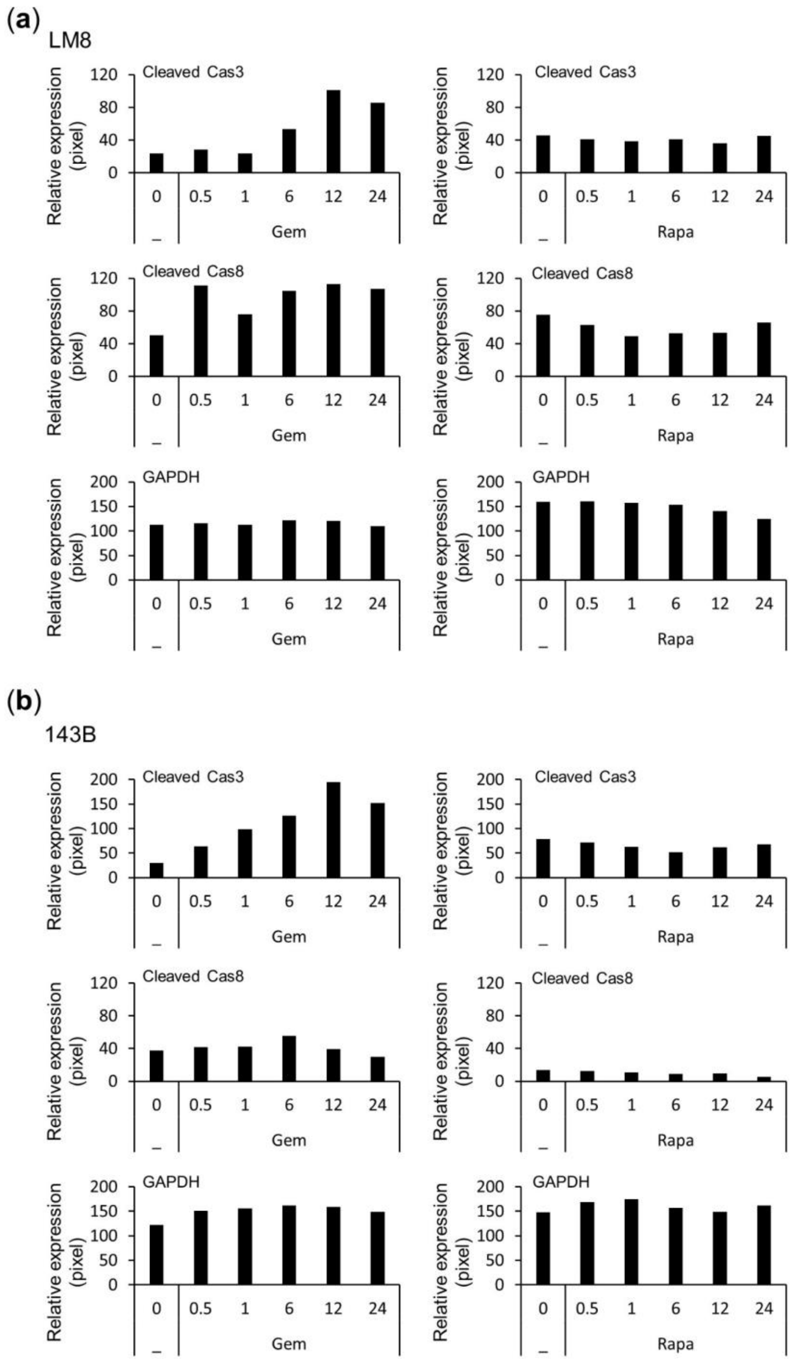

Figure S4. Quantification of cleaved caspase 3, cleaved caspase 8, and GAPDH bands in western blots depicted in Figure 2d. (a) LM8 cells. (b) 143B cells.

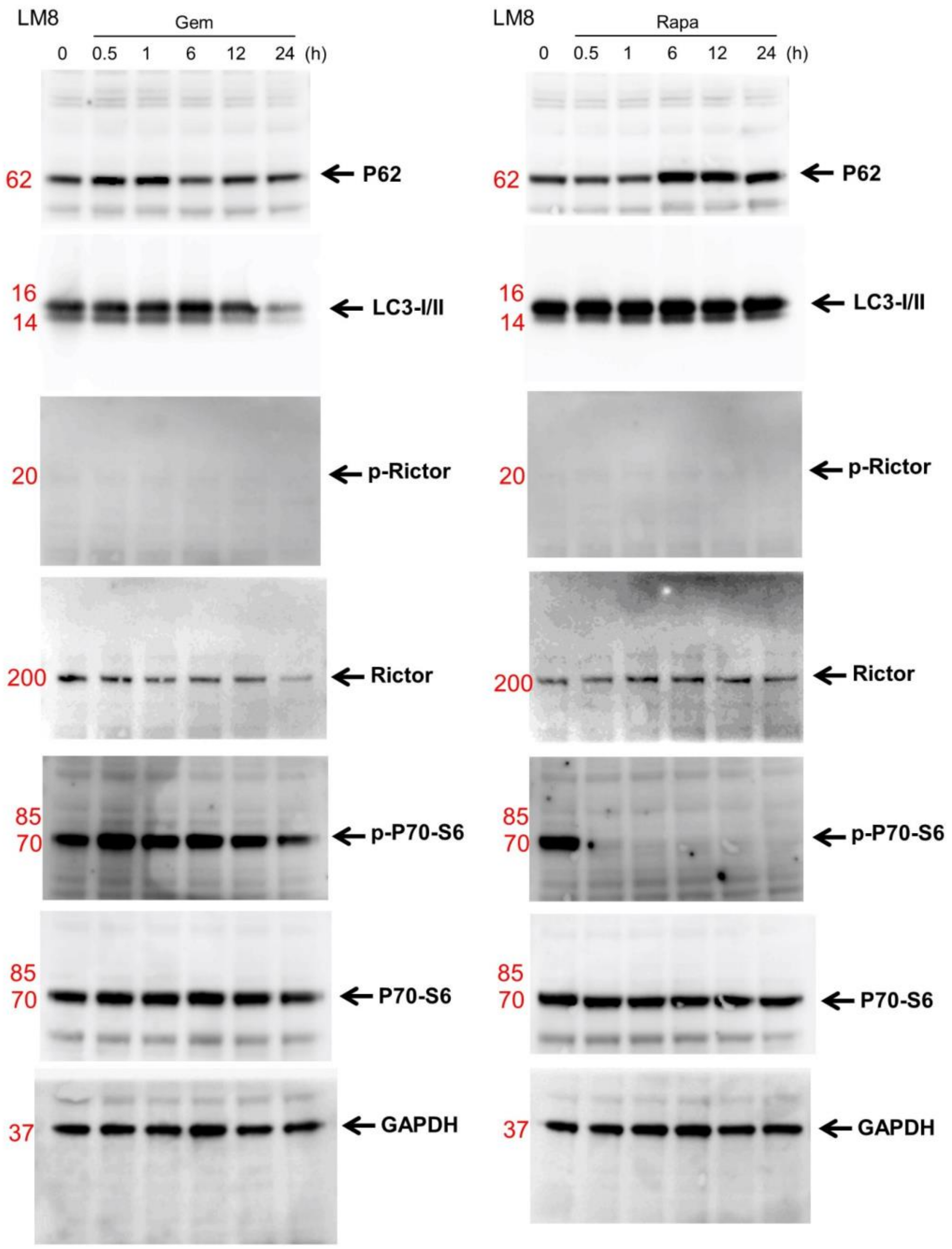

21 **Figure S5.** Example uncropped western blots depicting the expression of p62, LC3-I/II, p-RICTOR,
22 RICTOR, p-P70-S6, P70-S6, and GAPDH in LM8 cell lysates. These blots complement the cropped
23 blots in Figure 3a.

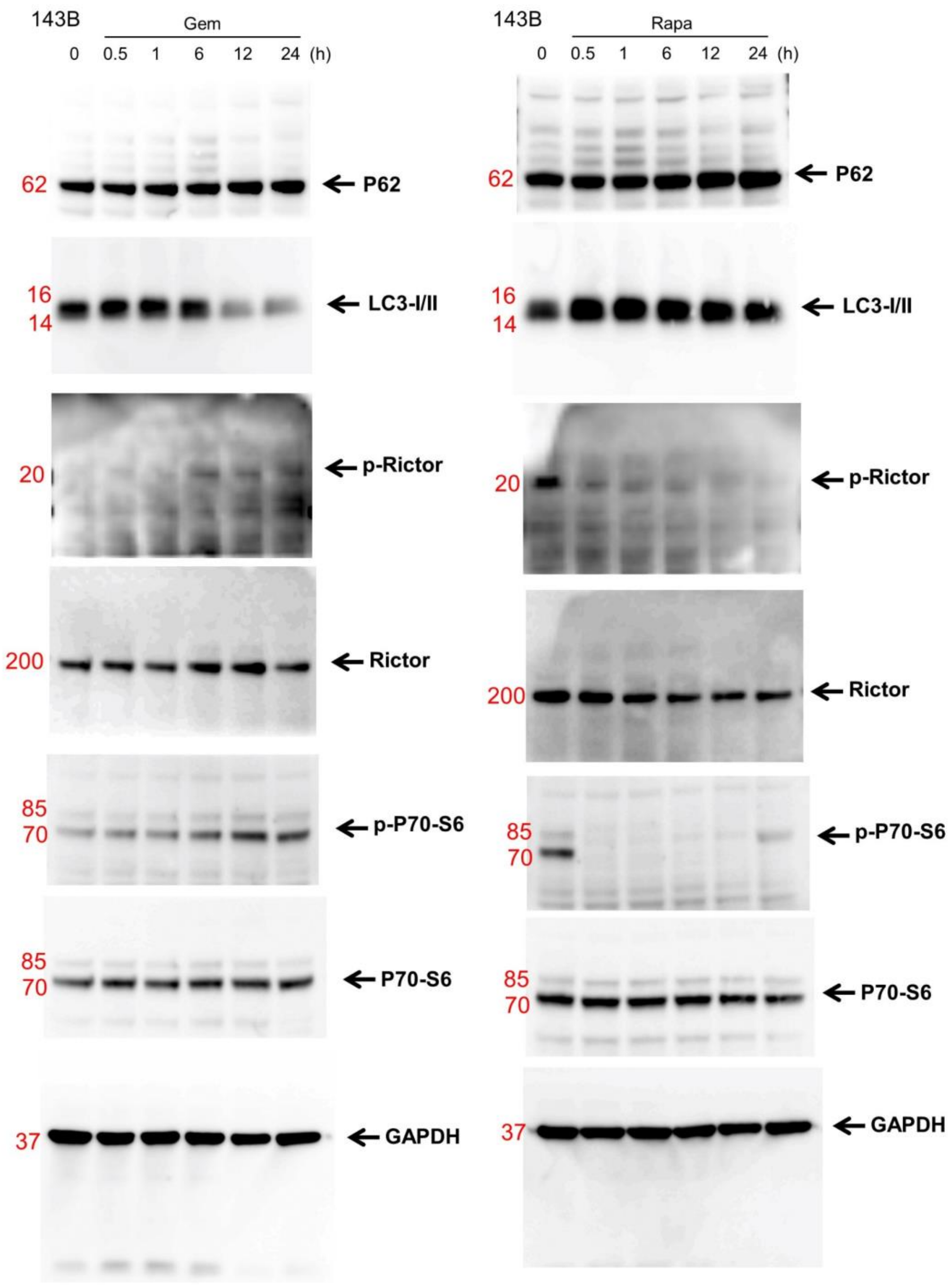

24 **Figure S6.** Example uncropped western blots depicting the expression of p62, LC3-I/II, p-RICTOR,
25 RICTOR, p-P70-S6, P70-S6, and GAPDH in 143B cell lysates. These blots complement the cropped
26 blots in Figure 3a.

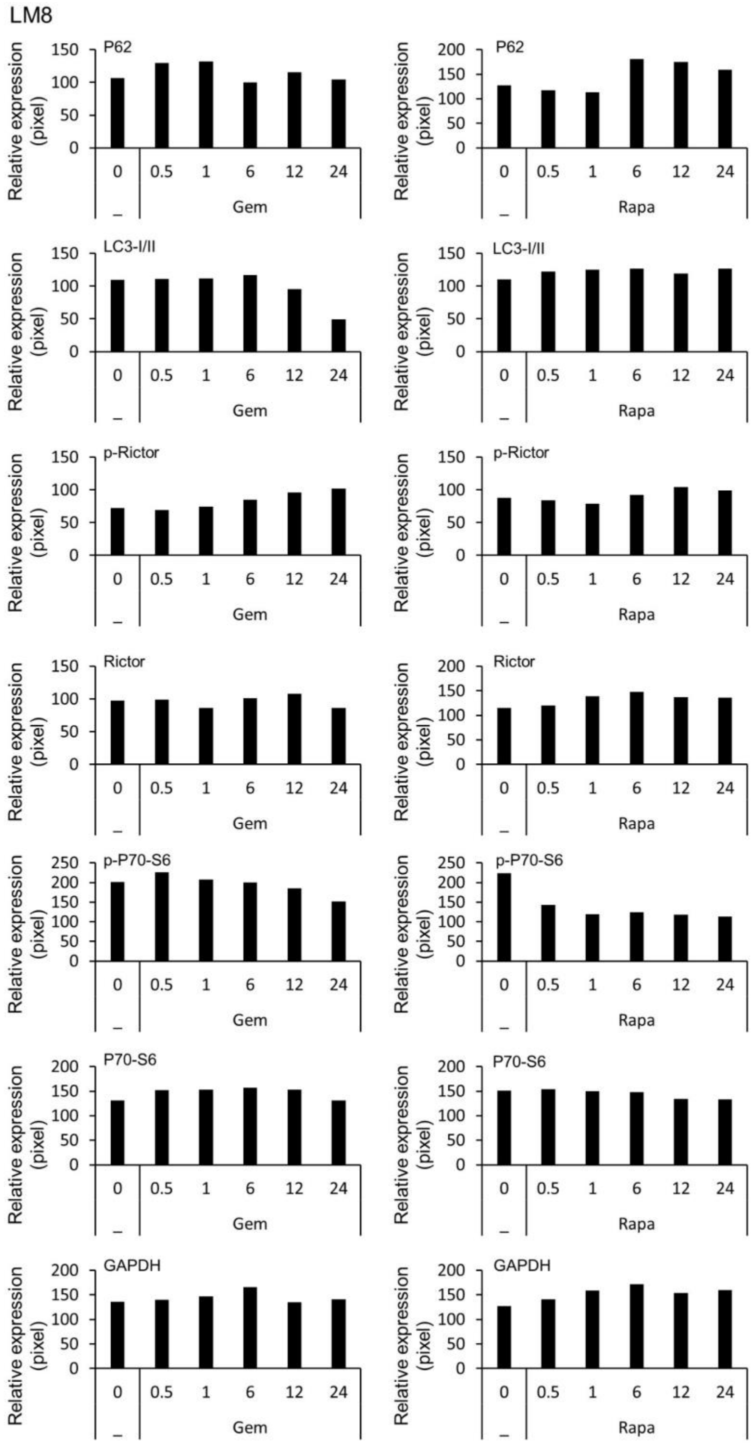

Figure S7. Quantification of western blot bands corresponding to p62, LC3-I/II, p-RICTOR, RICTOR, p-P70-S6, P70-S6, and GAPDH in LM8 cell lysates depicted in Figure 3a.

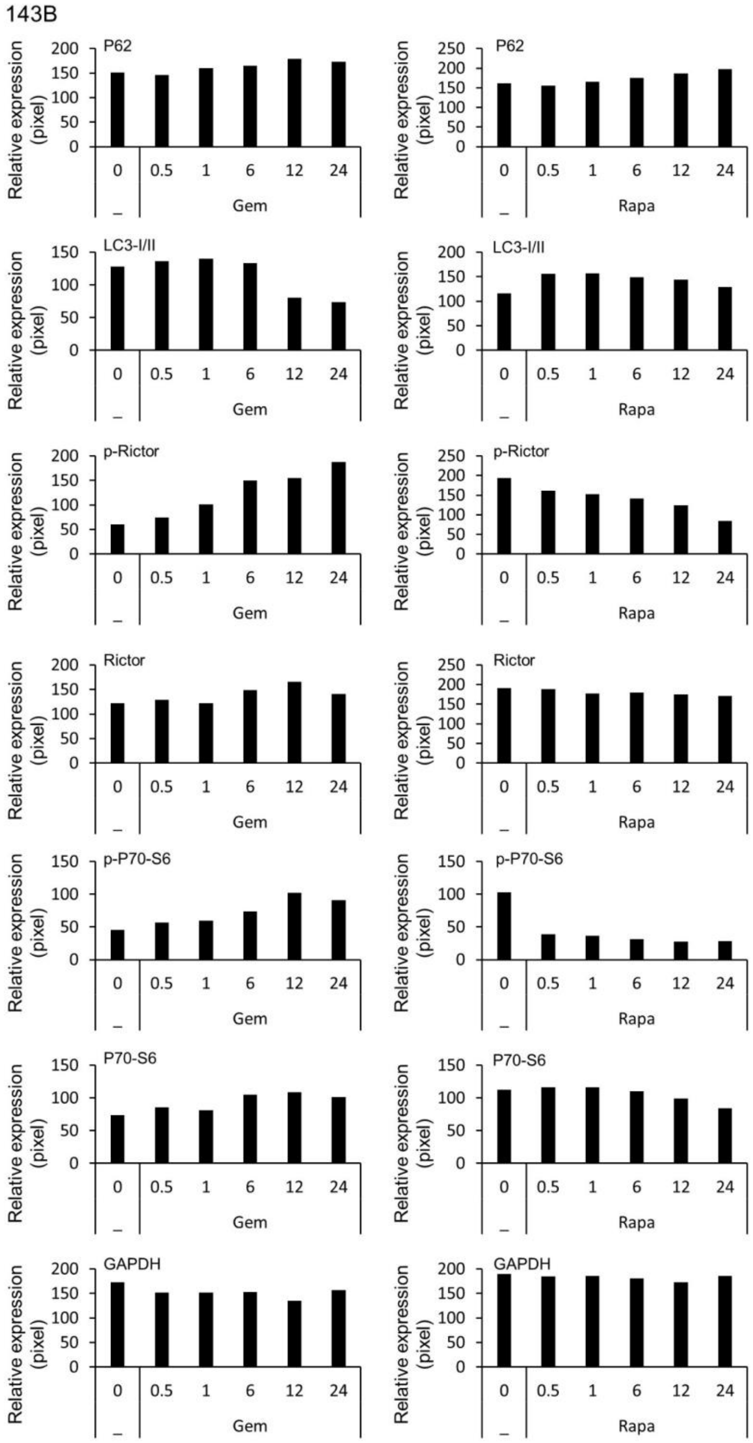

Figure S8. Quantification of western blot bands of p62, LC3-I/II, p-RICTOR, RICTOR, p-P70-S6, P70-S6, and GAPDH in 143B cell lysates depicted in Figure 3a.

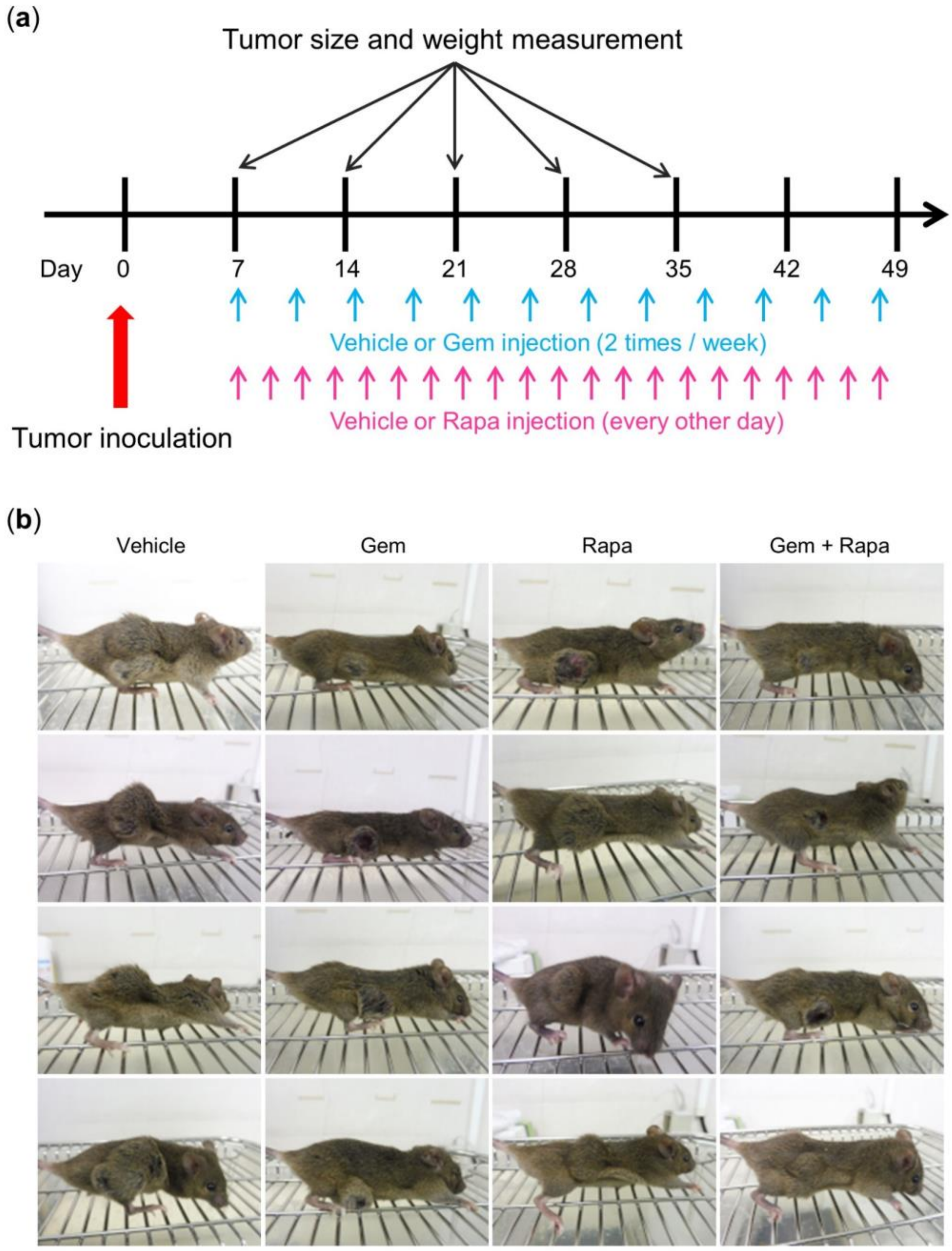

Figure S9. (a) Schematic diagram showing the experimental design for Gem and Rapa treatment of mice with allograft or xenograft osteosarcoma cell transplants. (b) Photos of tumor-bearing mice from Figure 4.

© 2020 by the authors. Submitted for possible open access publication under the terms and conditions of the Creative Commons Attribution (CC BY) license (http://creativecommons.org/licenses/by/4.0/).
